# Molecular profile of residual triple-negative breast cancer: opportunities for post-neoadjuvant therapeutic interventions

**DOI:** 10.1038/s41523-026-00964-7

**Published:** 2026-05-13

**Authors:** Nadine S. van den Ende, Marcel Smid, John W. M. Martens, Reno Debets, Agnes Jager, Carolien H. M. van Deurzen

**Affiliations:** 1 Department of Pathology, Erasmus University Medical Centre, Rotterdam, The Netherlands; 2 Department of Medical Oncology, Erasmus MC Cancer Institute, Erasmus University Medical Centre, Rotterdam, The Netherlands

**Keywords:** Biomarkers, Cancer, Genetics, Oncology

## Abstract

A subset of triple negative breast cancer (TNBC) patients shows resistance to standard neoadjuvant chemotherapy (NAC), resulting in high relapse and mortality risk. This highlights the need for predictive biomarkers and alternative treatment strategies. Targeted molecular profiling was performed on post-NAC resection specimens from 138 TNBC patients, diagnosed across multiple centers between 2013 and 2022, all exhibiting extensive poor response, defined as >50% residual tumor and the development of distant metastasis. Integrated immunohistochemistry and genomic analyses were conducted to identify potentially targetable alterations. Most post-NAC TNBCs (60%) were HER2-ultralow or HER2-low. Among 85 patients with successful DNA sequencing, 2640 variants were detected, with *TP53* mutations being most frequent (94%). Mutation count ranged from 3 to 1668 per patient (median *n* = 11). Several altered genes, including *ERBB2*, *BRCA1/2, PIK3CA, and RB1*, have been associated with favorable responses to targeted therapeutics in clinical trials. Moreover, 208 potential neo-peptide targets (median per patient *n* = 3) were detected across recurrently mutated genes such as *ATM, CREBBP*, *IRS2*, *KEAP1, MSH6, NOTCH1, NOTCH2, POLD1, TP53*, and *TSC2*. Molecular profiling of residual disease in extensively poor responding TNBC post-NAC revealed multiple potentially targetable variant, supporting the use of next-generation sequencing to guide personalized strategies for these high-risk TNBC patients.

## Introduction

Triple negative breast cancer (TNBC) is characterized by the absence of the estrogen receptor (ER), progesterone receptor (PR), and human epidermal growth factor receptor 2 (HER2) expression^1,2^. TNBC accounts for approximately 20% of all breast cancers and is clinically associated with higher rates of recurrence, earlier metastases, and poorer overall outcomes^3,4^. For the past several years, the standard of care for TNBC has been neoadjuvant chemotherapy (NAC), typically based on anthracyclines, taxanes, and often platinum agents^5,6^. Recently, pembrolizumab has been integrated for stage II disease and above^5^. Approximately half of the patients achieve a pathologic complete response (pCR) after NAC, which is associated with good long-term survival^5,7^. Most patients with residual disease have a partial therapy response, which is associated with either good or moderate clinical outcome, depending on pre-NAC clinical stage and amount of residual tumor^8,9^. However, a relatively small, but clinically relevant subgroup shows limited or no therapy response. These extensive poor responders represent a more aggressive subtype of TNBC, characterized by a high risk of early relapse and mortality, underscoring the urgent need for alternative treatment strategies in this subgroup.

Over the past years, considerable efforts have been directed towards developing targeted therapies for TNBC^6,10,11^. These approaches include immunotherapy, in the form of immune checkpoint inhibitors (ICI), agents targeting DNA damage response pathways, such as PARP inhibitors for patients with *BRCA1/2* mutations or homologous recombination deficiency, and antibody-drug conjugates (ADC)^12–14^.

In addition, advances in cancer immunology have underscored the critical role of the tumor microenvironment (TME) and tumor-specific antigens in shaping personalized therapeutic decision-making^6,15^. These tumor-specific peptides, commonly referred to as neo-peptides, are peptides derived from tumor-specific somatic DNA mutations^16,17^. Accumulating evidence indicates that the presence of these neo-peptides can influence tumor immunogenicity and the efficacy of ICIs in TNBC as well as other cancers^18,19^. Unlike self-antigens or peptides, neo-peptides are not subject to the central immune tolerance, enabling them to elicit potent and specific T-cell-mediated immune responses. Their unique tumor-restricted expression profile (less than 0.003% is shared in more than 5% patients) positions them as potential candidates for targeted immunotherapeutic interventions, including adoptive T-cell therapies^16,20,21^. Within TNBC, the high mutational burden provides a strong foundation for the generation of neo-peptides^18^.

Given the poor prognosis of TNBC patients with substantial residual disease after NAC and the limited effectiveness of current adjuvant therapies, there is an urgent need to optimize treatment strategies for this patient population. A deeper understanding of the molecular determinants may reveal actionable therapeutic targets. Therefore, this study aims to characterize the molecular profiles of residual tumors in high-risk TNBC patients to contribute to the development of more tailored therapeutic strategies.

## Results

### General patient and tumor characteristics

In total, post-NAC resection specimen from 138 poor responders were included, with a median patient age of 49 years old. Clinicopathologic variables are shown in Table 1. Following NAC, a higher proportion of patients exhibited a lower T stage compared to the pre-NAC clinical stage, indicating that at least some therapy response was achieved. In line with this, the post chemotherapy nodal stage (ypN) was less often positive compared to the clinical nodal stage (cN) (46% versus 55%). The most frequent histologic subtype was invasive no special type (NST) carcinoma (78%), followed by the metaplastic subtype (11%). Most tumors were grade 3 (86%), showed no angioinvasion (79%), were keratine 5 positive (74%), and had a density of tumor infiltrating lymphocytes (TILs) < 10% (105 out of 138; 76%). Regarding HER2 expression, most tumors were HER2-ultralow or HER2-low (60%).

**Table 1:** Clinicopathologic characteristics of TNBC patients with a poor response to NAC

Characteristics	Number of cases (*n* = 138)
Age at diagnosis	
Median in year (range)	49 (24–76)
Pre-NAC clinical tumor size (cT)	
≤2 cm (T1)	9 (6.5%)
>2 – ≤ 5 cm (T2)	60 (43.5%)
>5 cm (T3)	36 (26%)
T4	16 (12%)
Unknown	17 (12%)
Pre-NAC clinical node stage (cN)	
Negative	42 (30.5%)
Positive	76 (55%)
Unknown	20 (14.5%)
Post-NAC pathologic tumor size (ypT)	
≤2 cm (T1)	24 (17%)
>2 – ≤5 cm (T2)	52 (38%)
>5 cm (T3)	31 (23%)
T4	7 (5%)
Unknown	24 (17%)
Post-NAC pathologic node stage (ypN)	
Negative	26 (19%)
Positive	64 (46%)
Unknown	48 (35%)
Histologic subtype^a^	
No Special Type	107 (78%)
Lobular	8 (6%)
Metaplastic	15 (11%)
Other	6 (4%)
Unknown	2 (1%)
Histologic grade^a^	
2	17 (12%)
3	119 (86%)
Unknown	2 (1%)
Angioinvasion^a^	
Absent	109 (79%)
Present	26 (19%)
Unknown	3 (2%)
Keratin 5	
Negative	10 (7%)
Focally positive	20 (15%)
Positive	102 (74%)
Unknown	6 (4%)
HER2 status^a^ (IHC)	
HER2-negative	54 (39%)
HER2-ultralow	64 (46%)
HER2-low	19 (14%)
Unknown	1 (1%)
Mitotic count^a^	
Median (range)	20 (1–86)
Ki-67 expression^a^ (IHC)	
Median percentage (range)	60 (1–90)
Density of stromal TILs^a^ (IHC)	
Median percentage (range)	5 (1–65)
CD8 positivity (IHC)	
Median number of positive cells/mm^2^ (range)	124 (3–1473)
Treatment type	
Anthracycline based	14 (11%)
Anthracycline and taxane based	65 (52%)
Anthracycline, taxane and platinum based	34 (27%)
Taxane and platinum based	13 (10%)

^a^Analyzed on post-NAC surgical specimen

Treatment and outcome data was available for 126 patients (91% of complete cohort). Among those with available records, 14 patients (11%) received anthracycline-based treatment. Anthracycline- and taxane-based treatment was administered to 65 patients (52%), and 34 patients received anthracycline-, taxane- and platinum-based treatment (27%). In addition, 13 patients were treated with taxane- and platinum-containing chemotherapy (10%). At the time of inclusion, 12 out of all 126 patients (10%) were still alive. Using pathologically confirmed recurrences after post-NAC breast surgery as a proxy for disease-free survival (DFS), the median DFS was 18 months. The median overall survival (OS) was 23 months for the entire cohort.

Outcome data stratified by treatment regimen showed a median DFS of 12 months and OS of 23 months for anthracycline-based therapy; a median of 20 and 26 months respectively, for anthracycline and taxane-based therapy; 14 and 20 months for anthracycline, taxane, and platinum-based therapy; and 14 and 19 months for taxane and platinum-based therapy. Log-rank tests showed no significant differences in DFS and OS between the four different treatment arms (DFS *p* = 0.233; OS *p* = 0.246; Supplementary Fig. 1A, B).

To compare platinum-containing therapy versus no platinum-containing therapy, patients were divided into two groups: 47 (37%) received platinum and 79 (63%) did not. Log-rank tests showed no significant differences in DFS and OS between the groups (DFS *p* = 0.706; OS *p* = 0.185; Supplementary Fig. 1C, D).

### DNA sequencing quality and overall mutational burden

A total of 101 samples (73% of the complete cohort of 138 patients) could be sequenced, based on the availability of a sufficient tumor cell cellularity. Mean depth of coverage across all samples was 1405X (range 189X–2309X). After quality control for these 101 sequenced samples, three samples were excluded based on coverage <250X and 13 samples were excluded based on contamination (>1%), leaving 85 samples for genomic analysis.

The total number of mutations per patient ranged from 3 to 1668 (median *n* = 11), with multiple mutations affecting the same gene in some samples. There was one sample with 1668 mutations, suggesting microsatellite instability. The next highest sample contained 38 variants. The tumor mutational load within all 85 samples combined was 2713 variants, including recurrent mutations and regardless of their VAF. The VAF of the mutations ranged from 0.6% to 100% (median 8%). Within these 85 samples, a total of 288 genes were found to be mutated in at least one patient, of which 243 were mutated in only one patient.

### Characteristics of the variants

**Fig. 1:**
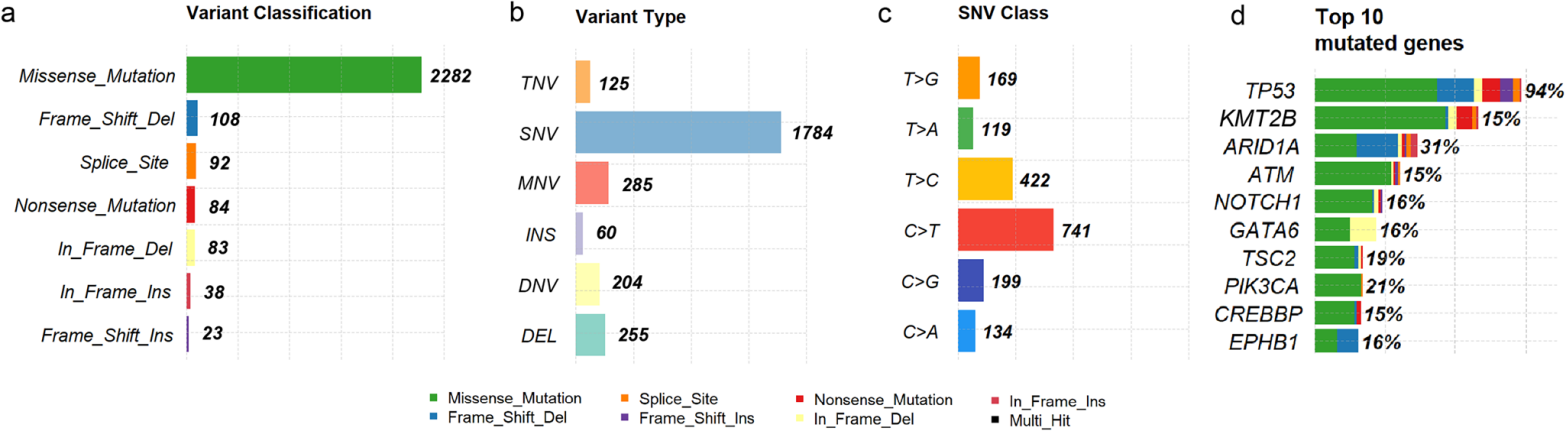
Variant characteristics of the identified mutations. Overview of the variant classification (**a**) and variant type (**b**), shown in relation to their frequency of occurrence across 85 TNBC cases (*x*-axis). **c** The type of SNV mutations and their frequencies. **d** Distribution of variant classifications across the top 10 mutated genes. TNV triple nucleotide variant, SNV single nucleotide variant, MNV multi nucleotide variant, INS insert, DNV double nucleotide variant, DEL deletion.

In total, 108 frameshift deletions, 23 frameshift inserts, 83 in frame deletions, 38 in frame inserts, 2282 missense, 84 nonsense, 3 promotor, and 92 splice mutations were identified. This highlights the differences among the observed mutations in terms of functionality, type of variant, single nucleotide variants (SNV) and other characteristics. Alterations were observed within the same gene and across multiple genes, with missense variants representing the most common type of mutation overall (Fig. 1a). From all the variant types, the SNV was the most common (Fig. 1b), and the C to T variant occurred most frequently among all SNV classes (*n* = 741; Fig. 1c). The distribution of variant types differed among the top 10 frequently mutated genes (Fig. 1d).

### Frequently mutated genes and mutual exclusivity

**Fig. 2:**
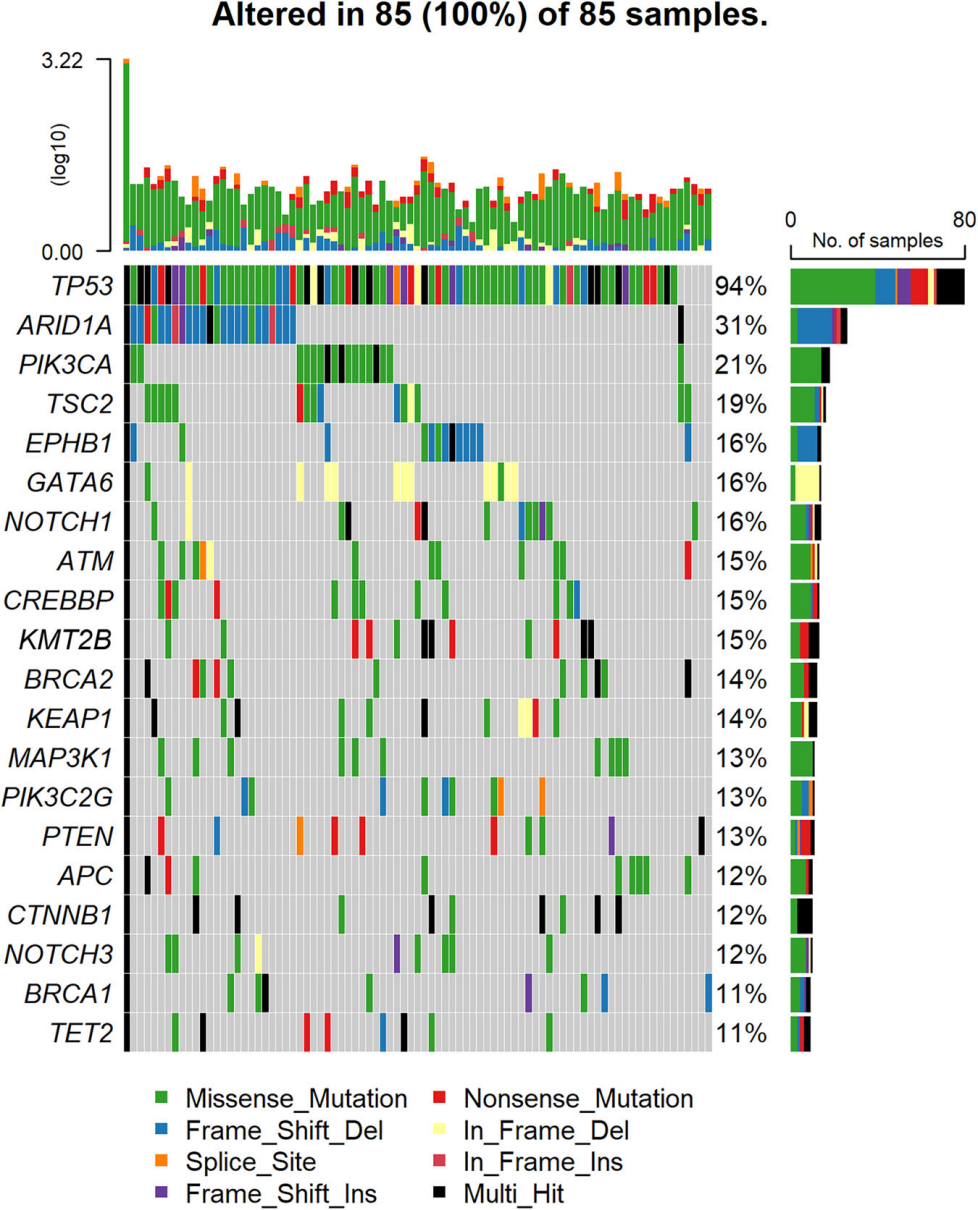
Alteration profile of the top 22 genes (mutated in >10% of cases) across 85 TNBC patients, highlighting the most frequently mutated genes. Each column on the x-axis represents one patient. Multi_hit indicates that more than one mutation was found in the same gene in the same sample.

*TP53* was the most frequently occurring mutated gene, in 94% of the patients, with VAFs ranging from 0.8% to 92% (median 51%; Fig. 2). The next most common alterations were found in the genes *ARID1A* (31%, VAF range 0.6–43%, median 6% with the majority frameshifts) and *PIK3CA* (21%, VAF range 0.9–90%, median 27%). Especially for *ARID1A*, the most variants appeared sub clonal with 77% (36 out of 47) showing a VAF < 10%. Since patients with an *ARID1A* mutation seemed to differ from those with a *PIK3CA* mutation, mutual exclusivity was tested among these frequently occurring mutations. This resulted in a *p*-value of 5.72E-05 for all alterations in these two genes and 0.017 for mutations with a minimum VAF of 2%, indicating mutual exclusivity between *ARID1A* and *PIK3CA*. The *PIK3CA* mutation occurred significantly more often in the metaplastic subtype compared to the other subtypes (40% versus 11%; *p* = 0.005). When comparing the mutational frequencies per gene between patients with or without platinum containing treatment, no significant differences were found after multiple testing (FDR p-0.1) Supplementary Fig. 2 illustrates the mutational profile according to treatment regimen. Moreover, the mutational burden was not significantly different between the patients with or without platinum containing treatment (median of 11 mutations in both groups; *p* = 0.26).

### Pathway-level analysis

**Fig. 3:**
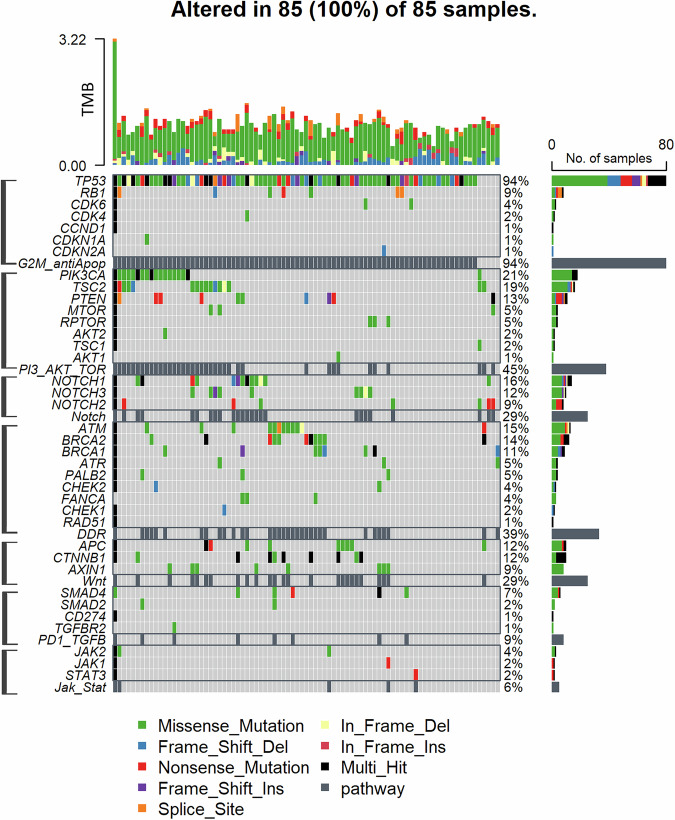
Oncoplot overview of the alteration profile in 85 TNBC patients, with alterations grouped by oncogenic pathway (indicated in gray). The top panel shows the total mutational load (TMB) per patient, with each column on the *x*-axis representing one patient.

The prevalence and spectrum of genetic mutations in key oncogenic pathways were analyzed to assess their clinical significance (Fig. 3). Genes were grouped according to their functional pathways, irrespective of their VAF. Mutation in genes from the PI3K/AKT/mTOR pathway were identified in 45% of the patients, with *PIK3CA* and *TSC2* being the most frequently altered (21% and 19%, respectively). Mutations in the DNA damage response pathway were detected in 39% of patients, with *ATM* and *BRCA2* as the most frequently mutated genes (15% and 14%, respectively). Alterations in the Wnt and Notch signaling pathways were each observed in 29% of the patients. These results underscored the heterogeneity of processes contributing to tumorigenesis and chemoresistance.

### Potential clinical implications based on tiers

Tumor mutation profiling was performed through annotating somatic alterations and mutational signatures using navify MP. The annotations incorporated curated information from previously published literature, databases, professional medical guidelines, approved drug labels, and clinical trial databases in accordance with ACMG/AMP variant classification^22^. When the mutations were subdivided into the classification in tiers, one gene was classified in the I-A category, four genes in the I-B category, 119 genes in the II-C category, and 24 mutated genes in the II-D category (Supplementary Table 1). The mutation in the tier I-A category was an amplification of the *ERBB2* gene, which was found in three patients. However, based on the immunohistochemistry (IHC) and in situ hybridization, these cases were considered as HER2 non-amplified. The mutations classified in tier I-B; *BRCA1*, *BRCA2*, *PIK3CA* and *RB1*, have previously been associated with clinical trial outcomes. Mutations in tier II-C either have approved drugs available for other cancers or have been described in literature in relation to clinical trial outcomes.

### Neo-peptides

**Fig. 4:**
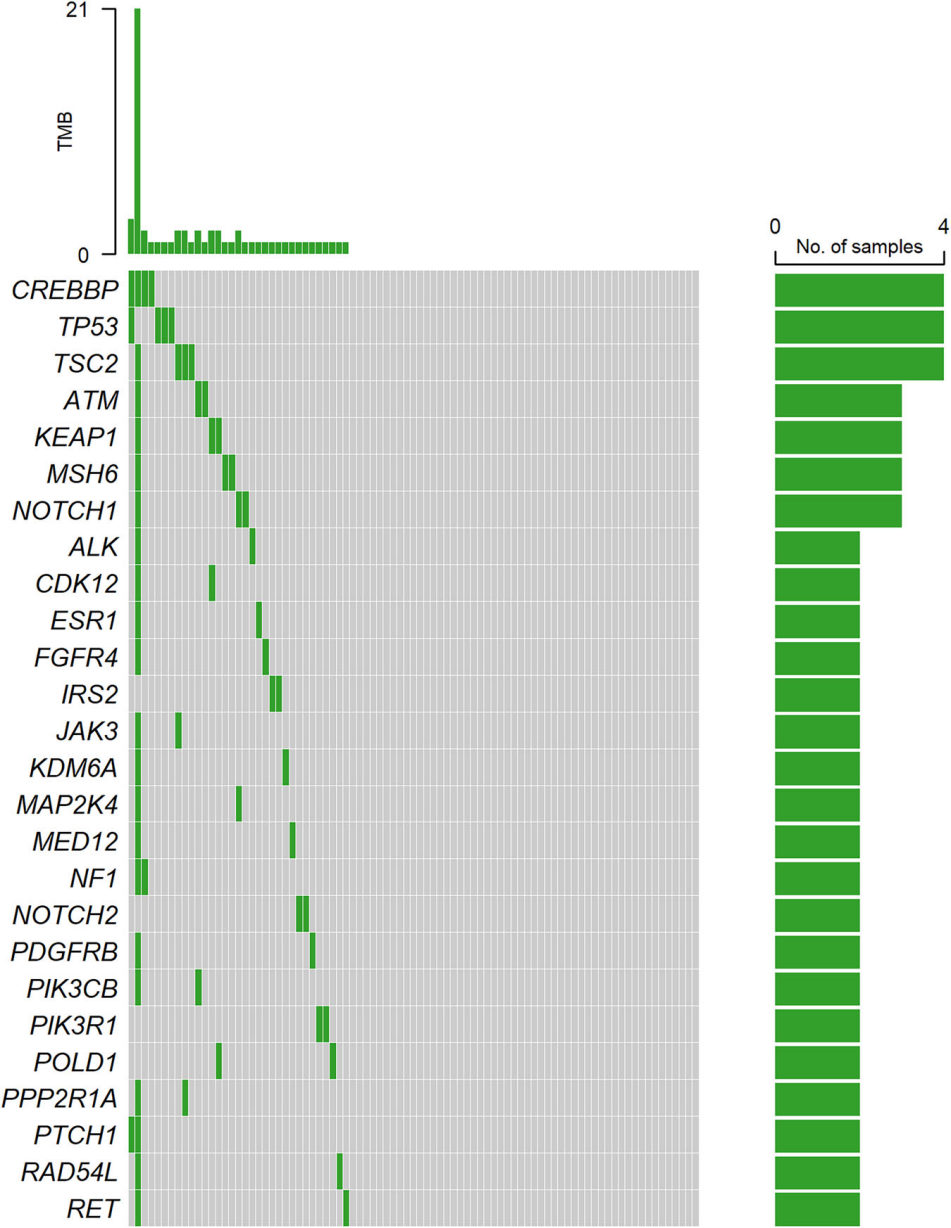
Oncoplot overview of the genes with the highest frequency of neo-peptides (≥2 cases) identified across 85 TNBC patients. The top panel illustrates the total mutational load (TMB) per patient for genes associated with neo-peptides. Each column on the x-axis represents one patient. *CREBBP*, *TP53*, and *TSC2* were identified as having the most neo-peptides within the gene (identified in 4 patients).

Neo-peptides potentially represent attractive targets for personalized immunotherapy. Therefore, we investigated the predicted binding strength of neo-peptides towards major histocompatibility complex, which is considered a critical first step in eliciting a T cell response. In fact, peptides with high predicted binding affinity are more likely to be effectively presented and recognized by T cells. Our results show that more than half of the patients have one or two predicted neo-peptides (58%; 49 out of 85). In total, 208 neo-peptides were found (median per patient *n* = 3; range 1–133). In addition, we observed that 76 non-recurrently mutated genes showed single neo-peptides, whereas 51 genes had two or more neo-peptides. Four genes, i.e., *ATM, KEAP1, MSH6*, and *NOTCH1*, each contained three predicted neo-peptides, and three other genes, i.e., *CREBBP, TP53*, and *TSC2*, even four neo-peptides (Fig. 4). Among all neo-peptides, six genes did exhibit recurrent mutations, these were *CREBBP, IRS2, MSH6, NOTCH2, POLD1*, and *TSC2*, each displaying neo-peptides in two separate patients. Furthermore, the number of predicted neo-peptides was strongly correlated with the number of mutations (*R* = 0.96).

### Comparison of mutational landscape with an external TNBC cohort

**Fig. 5:**
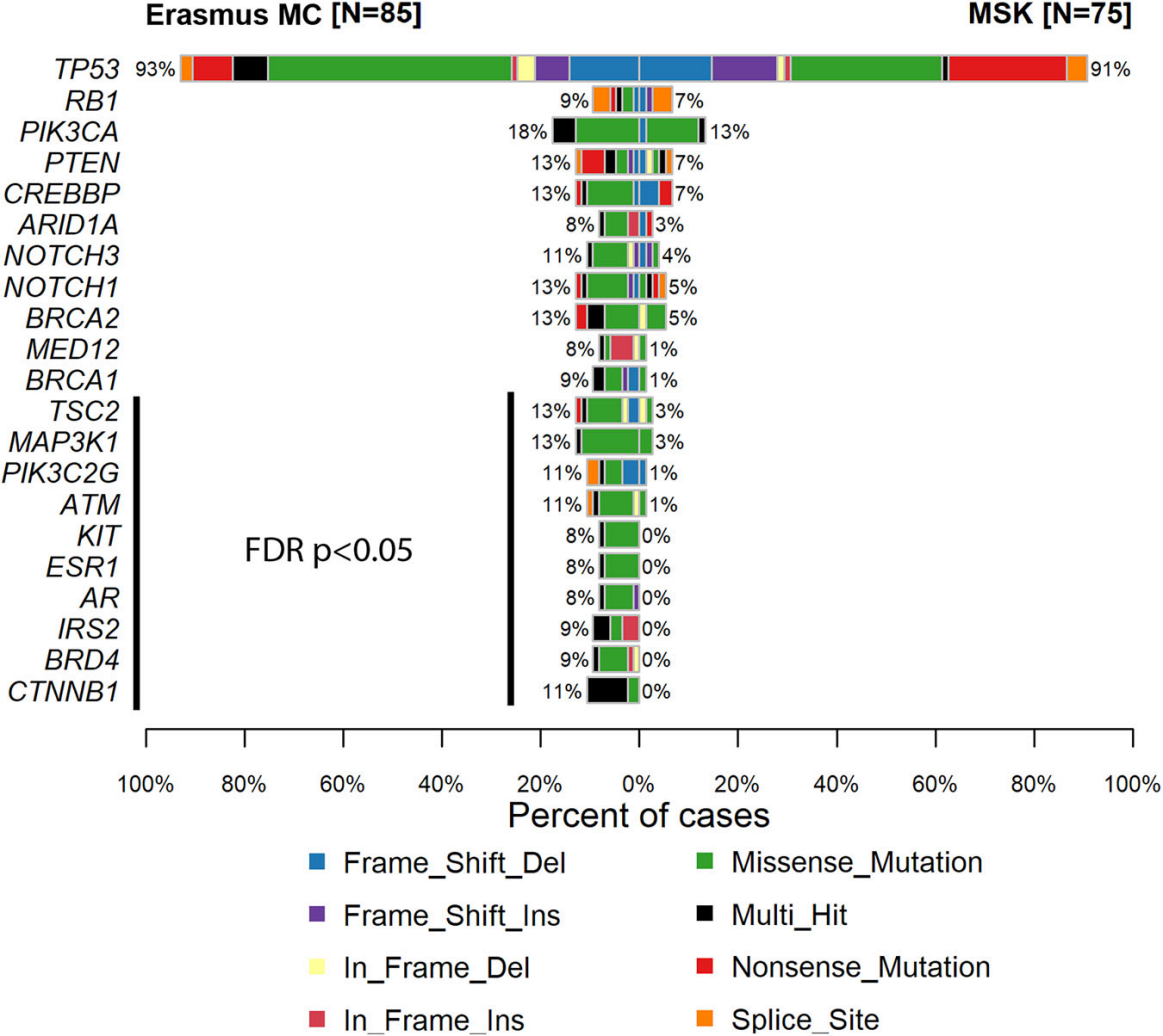
Comparison of the most frequently mutated genes in our cohort (Erasmus MC) and the MSK-IMPACT study. The vertical line indicates genes that are significantly enriched in our cohort compared to the external MSK cohort (FDR *p*-values < 0.05, see Supplementary Table 1 for all *p*-values). FDR false discovery rate.

We investigated whether the molecular characteristics underlying the aggressive biological behavior in our study cohort differed from those in a treatment-naïve primary TNBC population from the MSK-IMPACT study^23^. We restricted to genes with >2% VAF (to align with the external cohort) and to genes present in at least 10% of the samples within our cohort or the MSK study. This analysis revealed that ten genes were significantly more frequently mutated within our poor responder cohort compared with the reference TNBC population, including *CTNNB1*, *BRD4, IRS2, AR, ESR1, KIT, ATM, PIK3C2G, MAP3K1*, and *TSC2* (Fig. 5 and Supplementary Table 2).

## Discussion

In this study, we characterized the molecular landscape of residual tumor in TNBC patients with an extensively poor response to NAC, representing those patients with rapid progression and high mortality, despite the advances in post neoadjuvant treatment with capecitabine^24^. Therefore, there is an urgent clinical need to improve the outcome of these patients, which underscores the need to identify actionable therapeutic targets.

The tumors within this cohort were generally large, showed high proliferation activity, and contained a low number of TILs. Most cases were classified as either HER2-ultralow or HER2-low (60%). HER2-directed ADCs, such as trastuzumab deruxtecan, have recently demonstrated efficacy not only in HER2 + BC, but also in HER2-ultralow and HER2-low BC^25,26^. This indicates the potential benefit of adding this targeted HER2 therapy for this subgroup of patients.

In our cohort, the most frequently mutated gene was *TP53*, detected in 94% of all patients. This is in line with previous studies in TNBC and other cancer types^24,27,28^. *TP53* mutations are associated with genomic instability and loss of the tumor suppression function^29^. Other recurrently altered genes within our samples were *PIK3CA, RB1, ARID1A*, and *CTNNB1*, which have been previously reported at higher frequency in metaplastic BC^30–32^. This is in line with the relatively high proportion (11%) of metaplastic carcinomas and high levels of keratin 5 expression in this study.

Treatment heterogeneity may have affected the observed mutational profiles, since treatment is known to influence the mutational landscape (e.g., anthracyclines are known to induce distinct forms of DNA damage)^33^. Although our study was not powered to detect differences based on treatment regimen, we performed an exploratory analysis to assess whether mutational profiles differed across treatment groups. Neither gene-specific mutational frequencies nor overall mutational burden differed according to treatment. In addition, an explorative analysis of clinical outcomes by treatment regimen was performed. No significant differences in OS or DFS were observed between the treatment arms. However, these findings should be interpreted with caution since this study was not powered to detect differences between treatment groups. Furthermore, not all recurrences result in pathological confirmation, so our approximate DFS should be considered explorative, as this approach does not permit a complete assessment of DFS.

To identify alterations with potential therapeutic implications, we assessed the molecular findings according to their tier-based classification, which stratifies variants by level of clinical evidence and actionability. *ERBB2* was detected as a Tier I-A variant in three patients, although the exact status is questionable since pathological assessment did not show amplification. *BRCA1, BRCA2, PIK3CA*, and *RB1* were categorized as Tier I-B, consistent with known clinically actionable targets, and collectively identified in 15 patients. Approximately 15% of our poor responding patients carried *BRCA1/2* mutations, suggesting potential benefit from PARP inhibition^27^. This percentage is concordant with previous reports, although prior studies generally associated *BRCA* mutation TNBC with a good response to NAC^34^. From a functional perspective, several potential targetable events have been identified, including *ARID1A* inactivation, which has been linked to sensitivity with p300/CBP inhibitors in endometrial cancer^35^. Moreover, alterations within *TSC2* have been identified, which is a key regulator of the mTOR signaling pathway. Therefore, these patients might benefit from mTOR inhibitor therapeutics like everolimus, which showed promising results in breast cancer related studies^36^.

Furthermore, a notable proportion of frameshift mutations were observed. Such mutations are known to disrupt the normal reading frame of a gene, typically leading to the production of aberrant or truncated proteins. These altered proteins are often unstable or non-functional, as the shift in reading frame introduces premature stop codons or drastically changes the downstream amino acid sequence. This reduces the likelihood that these mutations will produce stable recognizable potential neo-epitopes capable of eliciting a robust immune response, thereby limiting their potential immunogenicity. On the other hand, disrupting genes like *TP53, PIK3CA, RB1, ARID1A*, and *CTNNB1* may create new immunogenic peptides (i.e., neo-epitopes). These neo-epitopes could consequently introduce the weak spot of the tumor, which could act as a therapeutic angle to treat the tumors. While the number of predicted neo-peptides was strongly correlated with the overall mutation count, our results suggest that mutation burden itself could serve as a pragmatic measure for immunotherapy selection rather than relying solely on neo-peptide prediction. The use of TMB as a promising tool to help define TNBC patients who are likely to benefit from ICI has already been reported in multiple studies^37,38^. Moreover, multiple recurrent strong-binding neo-peptides originated from *ATM, CREBBP, IRS2, KEAP1, MSH6, NOTCH1, NOTCH2, POLD1, TP53*, and *TSC2*. These findings suggest several candidate pathways and genes for targeted intervention, even though there is a lack of highly recurrent mutations across patients, these candidates could be used for neo-peptide-based therapeutic approaches, like ICIs or adoptive TCR-T cell therapy, within these patients with extensive residual disease. Binding affinity was assessed only for HLA-A02:01, one of the most prevalent HLA class I alleles in Western European populations and a common reference in neoantigen studies^39,40^. The predicted binding strengths of 9-mer peptides may differ substantially in individuals carrying other HLA alleles. However, this analysis was limited to in silico neo-peptide predictions restricted to a single HLA allele and did not include functional validation of the peptide immunogenicity. Therefore, these findings should be considered exploratory, and any conclusions regarding immunotherapeutic relevance and clinical applicability should be interpreted with caution.

The strength of this study is that it focuses on a unique national cohort of TNBC patients with minimal or no response to standard NAC, and progression to distant metastases. In contrast to previous studies, which generally classify all non-pCR patients as poor responders, our study specifically delineated a TNBC subgroup with a particularly aggressive phenotype with dismal prognoses and a high unmet clinical need for new treatment options. A focused molecular characterization of this highly chemoresistant TNBC subgroup revealed distinct genetic alterations compared to the general TNBC population, some of which represent previously unrecognized changes. Molecular profiling of post-NAC residual disease enables the identification of actionable alterations and resistance pathways that can guide personalized post-NAC therapeutic strategies. This includes targeted or immune-based therapies, rational combination approaches, or enrollment in biomarker-driven clinical trials.

In this cohort, the final analysis was restricted to 85 out of the initial 138 cases, after exclusion of tumors with low tumor cellularity or poor DNA quality. This may have led to selection bias by excluding cases with a slightly better treatment response or tumors exhibiting a more diffuse growth pattern, such as the lobular subtype. Another limitation of our targeted sequencing approach is the inability to distinguish between somatic and germline variants, due to the absence of matched normal samples. Variants were therefore presumed to be somatic unless otherwise documented. An implication of this limitation is the risk for false positives (germline mutations mistaken as somatic) or false negatives (true somatic mutations filtered out). Although population databases, allele frequency filtering, and predictive tools were applied, as described for the FoundationOne Analysis Platform, residual germline variants may still have contributed to an overestimation of TMB^41^. Additionally, this targeted approach limits the discovery of broader mutational events and neo-peptide diversity, restricting their utility in comprehensive biomarker discovery as well as underestimate the true mutational burden and omit potential immunogenic variants.

In conclusion, our results reveal a heterogeneous genomic landscape in TNBC patients with a poor response to NAC. Potentially targetable alterations were identified, indicating that molecular analysis of residual disease could have a role to guide and optimize post-neoadjuvant treatment strategies in this high-risk population.

## Methods

### General data acquisition

Using the Dutch Nationwide Pathology Databank (Palga), TNBC patients were selected for this retrospective study^42^. Formalin-fixed paraffin-embedded (FFPE) tissue blocks of post-treatment surgical resection specimens of TNBC patients with a poor response to standard NAC, diagnosed between 2013 and 2022, were collected from 27 hospitals across the Netherlands.

The study was performed according to the Declarations of Helsinki. The study protocol and data request were reviewed and approved by the scientific council and privacy committee of Palga (lzv2024-27). The Medical Research Ethics Committee of the Erasmus Medical Center concluded that this work was not subject to the Medical Research Involving Human Subjects Act (WMO; MEC-2021-0738). Patients who objected to the secondary use of residual material for research were not included in this study. Leftover patient material was coded and used in accordance with the Code of Conduct of the Federation of Medical Scientific Societies in the Netherlands^43^. Moreover, informed consent was waived as most of the patients were deceased at the start of the study, following guidance from the Privacy Knowledge Organization and legal counsel of the Erasmus Medical Center Rotterdam in compliance with the Institutional Review Boards and Ethics Committees of each institution (RC-0007199; Pursuant to Article 24 of the GDPR Implementation Act (UAVG) and Article 458 of the Medical Treatment Contracts Act (WGBO)).

A poor response was defined as 50% or more residual tumor in the surgical specimen, along with a pathology proven distant metastasis during follow-up through the end of the study inclusion period (2023). The percentage of residual disease was estimated by the pathologist based on the difference between the amount of tumor before and after NAC, and in this study, residual tumor burden corresponded to RCB-2 and RCB-3 disease. Patient and tumor characteristics were collected from the pathology reports. Clinical variables, treatment, and survival data was collected from the Dutch Cancer Registry. Clinical tumor size and nodal stage (cT and cN) were determined pre-treatment, while the pathologic tumor size and nodal stage (ypT and ypN) were assessed on post-treatment resection specimen. OS was defined as the time in months between the date of (needle biopsy) diagnosis and the date of death. DFS was not available in this nationwide cohort. However, we performed an exploratory analysis to approximate DFS, defined as the time between date of diagnosis and any pathologically confirmed recurrence of disease after post-NAC surgical tumor resection. Following Dutch guidelines, ER and PR negative is defined as less than 10% of positive tumor cells^43^.

### Central pathology review and immunohistochemistry

The pathology characteristics of the resection specimens were centrally reanalyzed which included histologic subtype, histologic grade, vascular invasion, keratin 5, HER2 status, Ki-67 expression, density of TILs, and density of CD8 positive T cells. For IHC, four µm sections of the whole tissues were cut (Micron HM340E) and mounted on Superfrost plus slides (Menzel-Glaser, Braunschweig, Germany). IHC staining was performed on the Ventana Benchmark ULTRA (Ventana Medical System Inc., ROCHE). Keratin 5 was scored as negative, focally positive (defined as intermediate staining in <10% of tumor cells) or widely positive (defined as intermediate to strong cytoplasmic and membranous staining in >10% of tumor cells)^44^. The HER2 score was determined based on the international ESMO and ASCO/CAP guidelines, where HER2-0 cases were further defined as either HER2-null (no expression) and HER2-ultralow (weak or incomplete membrane staining in ≤10% of invasive tumor cells). HER2-low breast cancer was defined by an IHC score of 1+ or 2+ without amplification^45^. Following the International Ki-67 in Breast Cancer Working Group, Ki-67 expression is determined based on the percentage of positive tumor cells within the complete tumor area^46^. The density of stromal TILs was scored manually on hematoxylin and eosin-stained whole slides, according to the recommendations of the International TILs Working Group^47,48^. The density of CD8 positive cells was scored using the open-source image analysis software Qupath, as a continuous variable based on the number of positive cells per mm^2^ within the tumor area^49^.

### DNA isolation and sequencing

Samples were preselected based on the presence of more than 20% tumor cell content within the whole tissue slide for inclusion into the genomic analysis. Two curls of 10 micron thickness were cut from the FFPE tissue blocks for DNA extraction, which was subsequently isolated and sequenced using the AVENIO Tumor Tissue CGP v1.0 Kit (Roche; for research use only; not for use in diagnostic procedures). The selected genes of interest were enriched using the AVENIO Tumor Tissue CGP v1.0 panel. This panel was designed to match the 324 gene FoundationOne® CDx Panel content and can detect genomic alterations (e.g., Single Nucleotide Variants (SNVs) and Insertions and Deletions (Indels)). Quality control measurements were performed using the 2100 Bioanalyzer (Agilent Technologies) and sequencing was performed on the Ilumina NextSeq 500.

After sequencing, variants were called using the AVENIO Connect Software v2.3.0 (Roche; for research use only; not for use in diagnostic procedures) on the FoundationOne® analysis platform. A minimum depth coverage of 250x was required, and no exclusion was performed in the first instance based on the variant allele frequency (VAF) in the tumor samples. To test for mutual exclusivity between somatic alterations, the Discrete Independence Statistic Controlling for Observations with Varying Event Rates (DISCOVER) method was used^50^. Final variant calls are determined through a series of quality control filters that exclude calls based on intrinsic sample noise, the expected noise level for a particular variant, and other known error modes (e.g., sequence homology). In addition, population SNVs are thoroughly filtered using the dbSNP and gnomAD databases, and with the use of a somatic-germline/zygosity algorithm the potential germline status was analyzed to allow for somatic variant calling and tumor mutational burden calculations^41^. Visualization of the mutated genes and variant information were created using the Maftools package and R (version 4.3.2)^51^. The mutational load was defined as the total number of variants detected per patient.

### Clinical variant interpretation using the Tier Classification

Clinical interpretation was performed using a tertiary analysis tool, the navify® Mutation Profiler (MP; Roche; version 2.5). Navify MP is an assay-agnostic interpretation software that uses the Association for Molecular Pathology (AMP) classification to establish which mutations are clinically actionable^52^. The variants were classified into tiers based on the level of evidence supporting their clinical significance. The classification of the tiers is as follows: I-A) Variants of Strong Clinical Significance supported by Agency or Medical Guideline Recommendation and High Evidence; I-B) Variants of High Clinical Significance based on Medical Guideline Recommendation or High Evidence within same tumor type; II-C) Variants of Potential Clinical Significance based on High Evidence in other tumor types or emerging evidence from clinical trials; II-D) Variants of Potential Clinical Significance based on preclinical or weak clinical evidence; III) Variants of Unknown Clinical Significance; IV) Benign or Likely Benign based on allele frequencies or lack of cancer association. Variants within the I-A, I-B and II-C tier are considered actionable.

### Neo-peptide analysis

Prediction of neo-peptides was performed using NetMHCpan-4.1 (https://services.healthtech.dtu.dk/services/NetMHCpan-4.1/)^53^, principally as described previously^15,54^. In brief, 17-mer peptides containing a mutated amino acid derived from a nonsynonymous mutation at the center position were run through the online server Net-MHC to predict binding strengths of all possible 9-mer peptides for HLA-A02:01 molecules. A peptide with an EL_Rank score <0.5 was considered a potential neo-peptide.

### Statistical analysis

Statistical analyses were performed using IBM SPPS Statistics version 26 and STATA (v13). The Pearson Chi-square or Fisher’s exact tests were used to investigate associations between categorical variables. For the continuous variables a Mann-Whitney U-test or the Kruskal–Wallis test was used in case of not normally distributed data. The association between mutational burden and treatment regimens was analyzed using the Mann-Whitney U-test. The survival outcomes (DFS and OS) between the treatment regimens were assessed using Kaplan-Meier estimators and differences were statistically compared using the log-rank test. A two-sided *p*-value below 0.05 was considered statistically significant.

## Supplementary information

Supplementary Data.

## Data Availability

The dataset generated and analyzed during the current study is not publicly available due to privacy regulations on the use of such data but are available from the corresponding author on reasonable request.
