# Supplementary material for: Molecular profile of residual triple-negative breast cancer: opportunities for post-neoadjuvant therapeutic interventions
Source: NPJ Breast Cancer. 2026 May 13;12:94. doi: 10.1038/s41523-026-00964-7 (PMC13392274; doi:10.1038/s41523-026-00964-7)
Supplement: Supplementary file 1 — Supplementary Data. [file 41523_2026_964_MOESM1_ESM.docx]

**
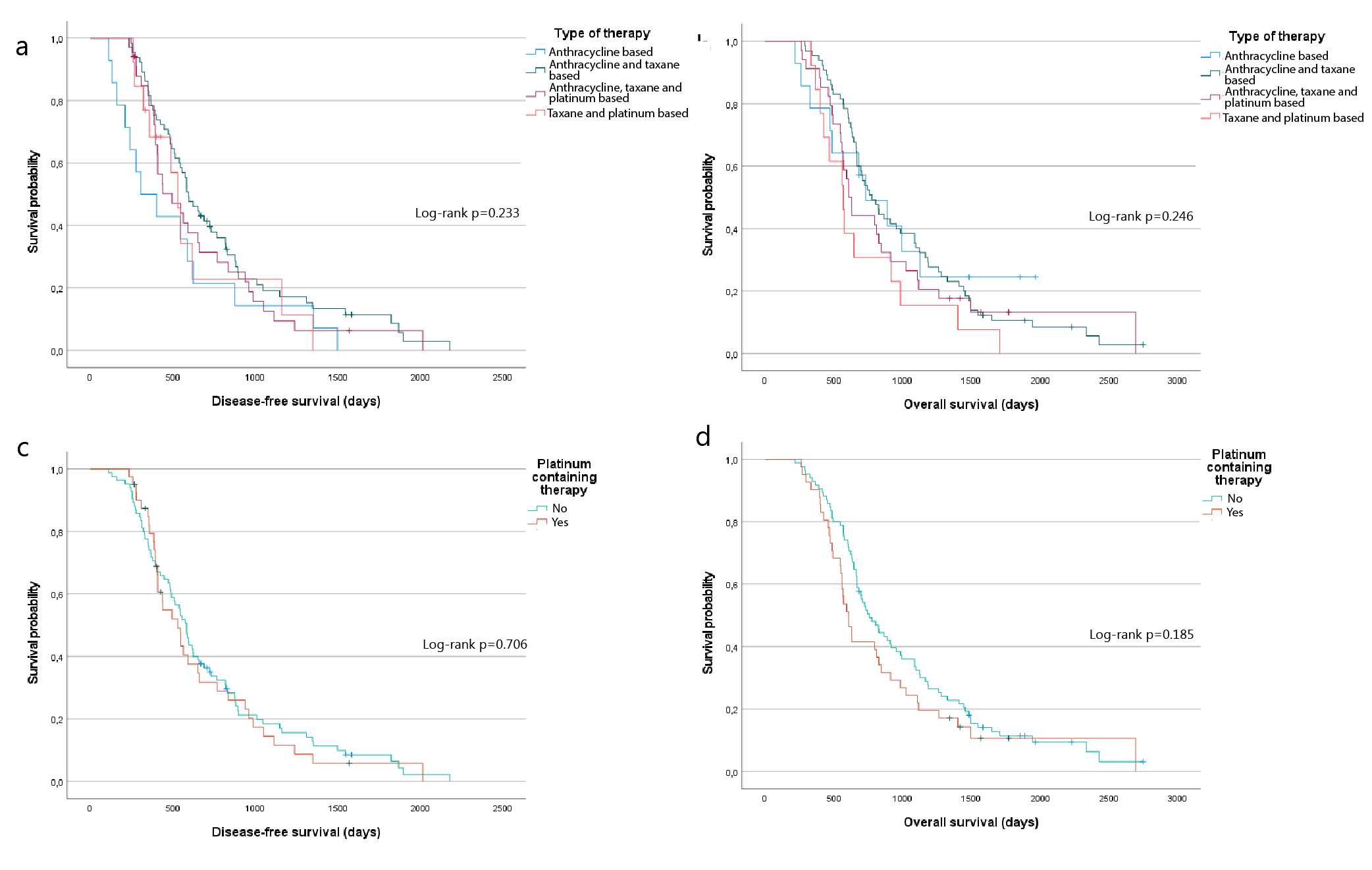
Supplementary figure 1.** *Kaplan Meier curves showing survival probability in days*. A) Approximate disease-free survival and B) overall survival across all treatment regimens. C) Approximate disease-free survival and D) overall survival for patients treated with or without platinum-containing therapy.

**
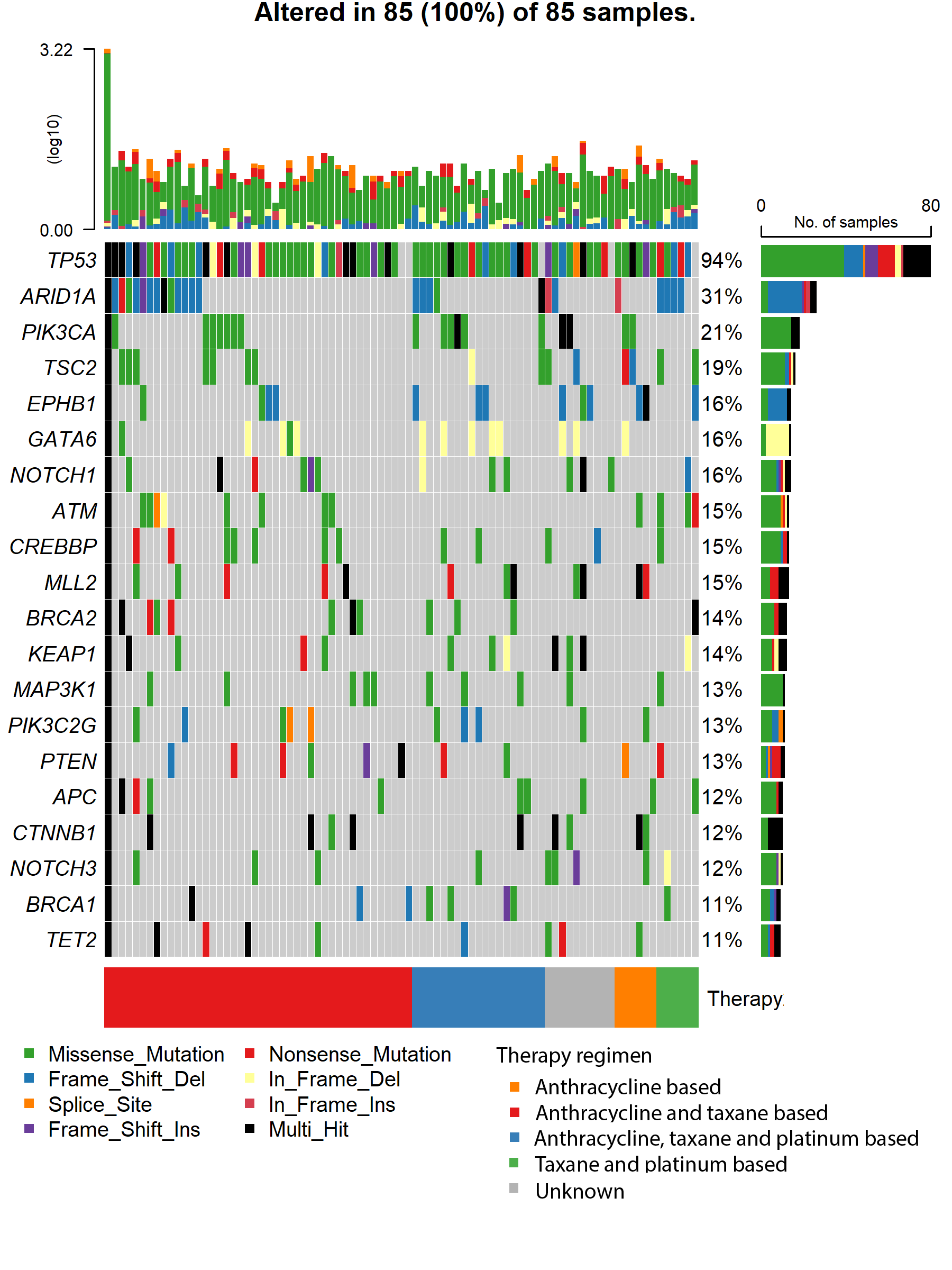
**

**Supplementary figure 2.** *Alteration profile of the top 20 most frequently mutated genes (mutated in >10% of cases) across 85 TNBC patients, stratified by treatment regimen*. Each column on the x-axis represents one patient. Multi_hit indicates that more than one mutation was found in the same gene in the same sample.

**Supplementary table 1**

Overview of the genes that were compared for the mutation status in our cohort and the mutation status in the MSK-IMPACT study, including statistical significance (p<0.05).

| **Tier I.A. gene mutation** | **Frequency** |
| --- | --- |
| ERBB2 | 3 |
|  |  |
| **Tier I.B. gene mutation** | **Frequency** |
| BRCA1 | 5 |
| BRCA2 | 3 |
| PIK3CA | 2 |
| RB1 | 5 |
|  |  |
| **Tier II.C. gene mutation** | **Frequency** |
| AKT1 | 3 |
| AKT2 | 6 |
| AKT3 | 12 |
| ALK | 1 |
| APC | 2 |
| ARID1A | 20 |
| ASXL1 | 1 |
| ATM | 2 |
| AURKA | 3 |
| BAP1 | 3 |
| BRAF | 6 |
| C8orf86::FGFR1 | 1 |
| CCND1 | 5 |
| CCND1 + FGF19 + FGF3 + FGF4 | 4 |
| CCND2 | 9 |
| CCND3 | 6 |
| CD274 | 5 |
| CDH1 | 2 |
| CDKN1B | 1 |
| CDKN2A | 11 |
| CDKN2B | 9 |
| CDKN2C | 1 |
| CHEK1 | 3 |
| CHEK2 | 1 |
| CIC::CIC | 1 |
| DDR1 | 4 |
| DDR2 | 13 |
| EGFR | 2 |
| ERBB2 | 2 |
| ERBB2 + ERBB3 | 1 |
| ERBB2 + PIK3CA | 1 |
| ERBB2 + TP53 | 2 |
| ERBB3 | 3 |
| EZH2 | 5 |
| FANCA | 7 |
| FANCC | 1 |
| FBXW7 | 4 |
| FGF19 | 9 |
| FGF19 + FGF3 + FGF4 + FGFR1 | 1 |
| FGF3 | 9 |
| FGF3 + FGFR1 | 2 |
| FGF4 | 9 |
| FGFR1 | 9 |
| FGFR1::RRAGC | 1 |
| FGFR2 | 5 |
| FGFR4 | 2 |
| FH | 1 |
| FLCN | 1 |
| FLT3 | 2 |
| HRAS | 5 |
| IDH1 | 1 |
| JAK1 | 1 |
| KDR | 2 |
| KEAP1 | 6 |
| KIT | 3 |
| KMT2D | 7 |
| KRAS | 10 |
| MAP2K1 | 2 |
| MAP2K4 | 1 |
| MAPK1 | 1 |
| MCL1 | 39 |
| MDM2 | 1 |
| MDM4 | 8 |
| MED12 | 1 |
| MET | 2 |
| MLH1 | 4 |
| MSH2 | 2 |
| MSH6 | 3 |
| MTAP | 7 |
| MUTYH | 2 |
| MYC | 37 |
| MYC::CLSTN3 | 1 |
| MYCN | 4 |
| NF1 | 6 |
| NF2 | 1 |
| NOTCH1 | 2 |
| NOTCH2 | 8 |
| NOTCH3 | 6 |
| NRAS | 1 |
| NTRK1 | 14 |
| NTRK2 | 1 |
| PDCD1LG2 | 5 |
| PDGFRA | 1 |
| PDGFRB | 1 |
| PIK3C2G | 5 |
| PIK3CA | 17 |
| PIK3CA + FGFR1 | 2 |
| PIK3CA + FGFR2 | 1 |
| PIK3R1 | 1 |
| POLD1 | 2 |
| PRKCI | 4 |
| PTCH1 | 1 |
| PTEN | 15 |
| PTEN + PIK3CA | 2 |
| RAD51C | 4 |
| RAD54L | 1 |
| RET | 2 |
| RICTOR | 2 |
| RNF43 | 1 |
| RPTOR | 4 |
| SDHB | 1 |
| SETD2 | 3 |
| SMARCA4 | 3 |
| SMARCB1 | 3 |
| SMO | 5 |
| SOX9 | 3 |
| SRC | 1 |
| STK11 | 5 |
| SUFU | 5 |
| TERT | 3 |
| TET2 | 4 |
| TP53 | 70 |
| TP53 + BRCA1 | 4 |
| TP53 + BRCA2 | 1 |
| TP53 + PTCH1 | 1 |
| TP53 + SMO | 4 |
| TP53 + SUFU | 5 |
| TSC1 | 2 |
| TSC2 | 3 |
|  |  |
| **Tier II.D. gene mutation** | **Frequency** |
| AR | 14 |
| BRCA1 | 2 |
| CD274 + JAK2 + PDCD1LG2 | 5 |
| CDK8 | 4 |
| CREBBP | 5 |
| CTNNB1 | 4 |
| EGFR + NF1 | 1 |
| EP300 | 3 |
| ESR1 | 1 |
| IKBKE | 9 |
| IRS2 | 2 |
| JAK2 | 5 |
| KDM5A | 14 |
| KDM6A | 2 |
| KMT2A | 1 |
| MAF | 1 |
| PIK3CA + MYC | 1 |
| RAC1 | 7 |
| RAD52 | 15 |
| SF3B1 | 1 |
| STAT3 | 1 |
| TP53 + BAP1 + MCL1 | 1 |
| TP53 + PTEN | 14 |
| TP53 + RB1 | 4 |

**Supplementary table 2**

List of gene mutations detected across all 85 TNBC patients, including the frequency and tier allocation.

|  | Hugo_Symbol | EMC | MSK | pval | or | ci.up | ci.low | adjPval |
| --- | --- | --- | --- | --- | --- | --- | --- | --- |
| 1 | CTNNB1 | 9 | 0 | 0.0036 | Inf | Inf | 1.8568 | 0.0429 |
| 2 | BRD4 | 8 | 0 | 0.0073 | Inf | Inf | 1.5875 | 0.0429 |
| 3 | IRS2 | 8 | 0 | 0.0073 | Inf | Inf | 1.5875 | 0.0429 |
| 4 | AR | 7 | 0 | 0.0148 | Inf | Inf | 1.3259 | 0.0429 |
| 5 | ESR1 | 7 | 0 | 0.0148 | Inf | Inf | 1.3259 | 0.0429 |
| 6 | KIT | 7 | 0 | 0.0148 | Inf | Inf | 1.3259 | 0.0429 |
| 7 | ATM | 9 | 1 | 0.02 | 8.6741 | 388.47 | 1.1525 | 0.0429 |
| 8 | PIK3C2G | 9 | 1 | 0.02 | 8.6741 | 388.47 | 1.1525 | 0.0429 |
| 9 | MAP3K1 | 11 | 2 | 0.0205 | 5.3772 | 51.583 | 1.1162 | 0.0429 |
| 10 | TSC2 | 11 | 2 | 0.0205 | 5.3772 | 51.583 | 1.1162 | 0.0429 |
| 11 | BRCA1 | 8 | 1 | 0.0371 | 7.6134 | 345.11 | 0.9805 | 0.0707 |
| 12 | MED12 | 7 | 1 | 0.0677 | 6.5795 | 302.83 | 0.8136 | 0.1185 |
| 13 | BRCA2 | 11 | 4 | 0.112 | 2.6236 | 11.829 | 0.734 | 0.168 |
| 14 | NOTCH1 | 11 | 4 | 0.112 | 2.6236 | 11.829 | 0.734 | 0.168 |
| 15 | NOTCH3 | 9 | 3 | 0.1403 | 2.8251 | 16.86 | 0.6703 | 0.1964 |
| 16 | ARID1A | 7 | 2 | 0.1751 | 3.2542 | 33.094 | 0.5937 | 0.2298 |
| 17 | CREBBP | 11 | 5 | 0.2908 | 2.0719 | 8.0031 | 0.6248 | 0.3393 |
| 18 | PTEN | 11 | 5 | 0.2908 | 2.0719 | 8.0031 | 0.6248 | 0.3393 |
| 19 | PIK3CA | 15 | 10 | 0.517 | 1.39 | 3.7247 | 0.5397 | 0.5714 |
| 20 | RB1 | 8 | 5 | 0.5752 | 1.4512 | 5.9156 | 0.3968 | 0.604 |
| 21 | TP53 | 79 | 68 | 0.7733 | 1.3528 | 5.127 | 0.3693 | 0.7733 |

pval is Fisher’s exact test

adjPval = Bonferroni Holm FDR
